# Hypoxia Enhances the Senescence Effect of Bortezomib—The Proteasome Inhibitor—On Human Skin Fibroblasts

**DOI:** 10.1155/2014/196249

**Published:** 2014-01-29

**Authors:** Rafał Krętowski, Małgorzata Borzym-Kluczyk, Marzanna Cechowska-Pasko

**Affiliations:** Department of Pharmaceutical Biochemistry, Medical University of Bialystok, Mickiewicza 2A, 15-222 Bialystok, Poland

## Abstract

The 26S proteasome inhibitor, bortezomib, selectively induces apoptosis in some cancer cells. However, the nature of its selectivity remains unknown. The study presented here provides novel information on cellular effects of bortezomib in normal fibroblasts. We have found that in normoxic conditions the percent of apoptotic cells did not change significantly, independently on incubation time and examined concentration of bortezomib (25 nmol/L or 50 nmol/L). In hypoxic conditions we did not observe any effect of bortezomib on apoptosis of fibroblasts incubated for 24 h and 48 h in comparison to control. Only in the case of fibroblasts incubated for 12 hours in hypoxia significant increase in apoptosis, dependent on concentration of bortezomib, was observed. Our study has shown that bortezomib causes a time-dependent increase in senescence of normal fibroblasts, especially of these incubated in hypoxic conditions. Moreover, we demonstrated that oxygen regulated protein 150 (ORP150) expression was induced in fibroblasts in hypoxia conditions only, suggesting that this protein may play an important role in the cytoprotective response to environmental stress.

## 1. Introduction

The endoplasmic reticulum (ER) is the site of synthesis, maturation, folding, and oligomerization of newly synthesized proteins, such as secretory and membrane proteins. Moreover, the ER plays a key role in protein homeostasis. The folding process is regulated by a group of proteins, referred to as ER chaperones [1]. One of them is oxygen regulated protein 150/glucose regulated protein 170 (ORP150/GRP170) alternatively known as HSP12A [2]. It was originally identified in cultured astrocytes exposed to hypoxia. The ORP150 is a part of the ER machinery that assists in the assemble and folding of secretory and membrane proteins [2, 3]. Its expression is upregulated in hypoxia, serum starvation, glucose deprivation, ischemia, and treatment with tunicamycin or 2-deoxyglucose. The ORP150 plays a cytoprotective role for the maintenance of cellular viability [3].

Many stress conditions, such as hypoxia or nutrient deprivation, slow down the folding process and cause accumulation of unfolded/misfolded proteins in the cell [4–6]. Hypoxia is a factor inducing ER stress, although the underlying mechanism is unknown. Yoshida [7] suggested that during this process glycolytic enzymes are induced to sustain ATP production and then cells consume glucose. Perhaps a decrease in glucose concentration induced by hypoxia inhibits N-glycosylation, leading to ER stress [7].

The accumulation of unfolded proteins activates the so-called “unfolded protein response” (UPR), which involves at least four types of reaction: general translational attenuation (phosphorylation of the translation initiation factor 2); increased folding capacity (upregulation of ER chaperones); enhanced ER-associated degradation of misfolded and unfolded proteins; and, if the stress is severe and unsolvable, apoptosis [4–6].

Misfolded and unfolded proteins are returned from the ER to the cytosol and degraded in the proteasome [8]. The ubiquitin proteasome pathway is responsible for cell quality control by eliminating defective proteins from the cytosol and endoplasmic reticulum. Proteins are degraded by the ubiquitin proteasome pathway via two distinct steps: the covalent attachment of multiple monomers of ubiquitin molecules to a protein substrate and degradation of the tagged protein by the 26S proteasome [8, 9].

Bortezomib (([(1*R*)-3-methyl-1-({(2*S*)-3-phenyl-2-[(pyrazin-2-ylcarbonyl) amino]propanoyl}amino)-butyl]boronic acid), velcade, formerly known as PS-341) is a competitive inhibitor of 20S proteasome activity in whole cells. It is used in the treatment of multiple myeloma and some forms of non-Hodgkin's lymphoma [8, 9]. Bortezomib inhibition of the proteolytic activity of the 20S proteasome has been shown to induce proapoptotic ER stress and inhibit proteasome degradation of I*κ*B, an inhibitor of nuclear factor-*κ*B (NF-*κ*B) in the cancer cell [8–10].

The inhibition of the proteasome results in many toxic effects, including the accumulation of unfolded and damaged proteins [11–13]. In response to proteasome inhibition, the cell induces specific protective mechanisms, including the unfolded protein response [11], autophagy [14, 15], and, if the damage is severe, apoptosis [11, 16].

It is known that apoptosis plays the major role in control of cancer development. An organelle that can induce apoptosis when stressed is the ER. In fact, cells encounter multiple apoptotic stimuli during cancer progression, including nutrient deprivation or hypoxia. Accordingly, it was suggested that the well-documented antiapoptotic potential of chaperons may play a critical role in suppression of apoptosis in cancer cells [1, 8]. Recently, the attention of the scientists has shifted towards a novel role of cell senescence in control of cancer development, and with this shift our view on the role of chaperons in cancer has also evolved towards appreciation of the major role of chaperons in regulation of the senescence program [17].

Senescence can be generally characterised as a cellular stress response. This is a signal transduction process that leads to an irreversible growth arrest of cells in the G1 cell cycle phase [18]. Our knowledge about cell senescence occurring *in vivo *and, most importantly, as a desired result for cancer treatment, is very limited and a rather new field of research. Senescence was originally applied to the irreversible growth arrest of cells after prolonged proliferation under *in vitro *cell culture conditions. Now it has been extended to the irreversible proliferation arrest of cells caused by various stresses, including oxidative damage, telomere dysfunction, DNA damage, and oncogene induced senescence as well [19–24]. Tumour cells are exposed to many different—external as well as internal—sources of stress; therefore the induction of senescence constitutes an important block to tumour progression [18]. Senescence is a potent anticarcinogenic program and the process of neoplastic transformation involves series of events that allow cells to bypass senescence by inactivation of senescence associated pathways. Still, many tumour cells have retained the capacity to senesce in response to external stress stimuli. Most conventional anticancer therapies activate DNA damage signaling pathways, which aim to induce primarily apoptotic cell death but often treated cells do not die by apoptosis but rather undergo growth arrest or senescence. It is not fully understood currently which specific signals cause cells to undergo either senescence or initiate apoptosis [18]. Senescence runs parallel with an accumulation of damaged proteins at the molecular-cellular level. The attenuation of molecular chaperone inducibility and the simultaneous accumulation of damaged proteins raise the possibility that preservation of protein homeostasis is a major determinant in the occurrence and duration of cellular senescence [25].

The cellular and molecular effects of the proteasome inhibitor—bortezomib—on human skin fibroblasts are as yet poorly characterised. Its participation in senescence process remains unclear. We decided to study the effect of bortezomib on apoptosis and senescence of human skin fibroblasts incubated in hypoxia in comparison to normoxic conditions. We investigated the effect of bortezomib on the activity of senescence marker SA-*β*-galactosidase and induction of ORP150 chaperon in normal fibroblasts incubated in hypoxic and normoxic conditions and its correlation with apoptosis of these cells.

## 2. Materials and Methods

### 2.1. Reagents

Dulbecco's modified Eagle's medium (DMEM), containing glucose at 4.5 mg/mL (25 mM) with Glutamax, penicillin, streptomycin, and trypsin-EDTA were provided by Invitrogen (San Diego, USA), passive lysis buffer by Promega (Madison, USA), FBS Gold by Gibco (USA), BCA Protein Assay Kit by Thermo Scientific (USA), PE Annexin V Apoptosis Detection Kit I by BD Pharmingen (USA), Senescence Detection Kit by bioVision (USA), Sigma-Fast BCIP/NBT reagent, and alkaline phosphatase-labelled anti-mouse immunoglobulin G (IgG) by Sigma (St. Louis, USA). The monoclonal antihuman ORP150 antibody was purchased from IBL (Gunma, Japan) and anti-HIF-1*α* antibody from BD Transduction Laboratory (USA).

### 2.2. Cell Cultures

Normal human skin fibroblasts cell line (CRL1474) were obtained from American Type Culture Collection (ATCC). Cells were maintained in high glucose DMEM supplemented with 10% heat-inactivated foetal bovine serum GOLD (FBS GOLD), 2 mM L-glutamine, penicillin (100 U/mL), and streptomycin (100 *μ*g/mL). Cells were cultured in Falcon flasks (BD) in a 5% CO_2_ incubator (Galaxy S+; New Brunswick), at 37°C. Subconfluent cultures were detached with 0.05% trypsin 0.02% EDTA in calcium-free phosphate-buffered saline (PBS) and counted in cells counter Scepter (Millipore).

### 2.3. Cell Viability

Cell viability was measured according to the method of Carmichael et al. [26] using 3-(4,5-dimethylthiazol-2-yl)-2,5-diphenyltetrazolium bromide (MTT). Briefly, cells were seeded in 24-well plate at a density of 5 × 10^4^ per well. Confluent cells, cultured for 12 h, 24 h, and 48 h in normoxic conditions with different concentrations of bortezomib, were washed three times with PBS and then incubated with 1 mL of MTT solution (0.25 mg/mL in PBS) for 4 h at 37°C in 5% CO_2_ in an incubator. The medium was removed and 1 mL of 0.1 mol/L HCl in absolute isopropanol was added. Absorbance of converted dye in living cells was measured at wavelength of 570 nm. The viability of fibroblasts cultured in hypoxic conditions was calculated as percentage of control cells, incubated in normoxia. All the experiments were done in triplicate in at least three cultures.

### 2.4. Induction of Hypoxia in Cell Cultures

The cells (2.5 × 10^5^ in 2 mL of medium) were seeded in six-well plates and incubated until they achieved confluence (about 48 h). The high glucose DMEM was removed and replaced with 2 mL of the same fresh medium with 25 nmol/L, 50 nmol/L or without bortezomib. Control cell cultures were kept in normoxic conditions whereas the test cells were incubated in hypoxic conditions. Hypoxia was evoked by 12 h, 24 h, and 48 h incubation of cells in atmosphere containing a reduced to 1% oxygen concentration in hypoxia chamber (Galaxy 170R; New Brunswick an Eppendorf Company).

After incubation the culture media were removed, the cell layers were washed with PBS and submitted to the action of lysis buffer for determination of ORP150/GRP170 expression and protein assay. It allowed the separation of cells and extracellular matrix from the bottom of culture vessels and their suspension in the buffer. The cells incubated on six-well plates were detached with trypsin and analyzed by flow cytometry method.

### 2.5. Detection of Apoptosis

The cells (2.5 × 10^5^ in 2 mL of medium) were seeded in six-well plates and incubated until they achieved confluence. The fibroblasts were incubated in the high glucose DMEM in hypoxic and normoxic conditions with 25 or 50 nmol/L of bortezomib. The incubation was continued for 12 h, 24 h and 48 h. The cells were trypsinised and resuspended in DMEM and then in binding buffer. Cells were stained with FITC Annexin V and PI for 15 min at room temperature in the dark following the manufacturer's instructions (FITC Annnexin V apoptosis detection Kit I). Flow cytometry was performed using a FACSCanto II cytometer (Becton Dickinson). Data were analysed with FACSDiva software and dead cells were excluded based on forward- and side-scatter parameters.

### 2.6. Detection of SA-*β*-Galactosidase Expression

For cytochemical of SA-*β*-galactosidase staining 2.5 × 10^5^ cells in 2 ml of growth medium were seeded in Petri dishes (9.5 cm^2^) and incubated for 24 h in the high glucose DMEM. In this conditions they reached 70–80% confluence. Cells were incubated in the high glucose DMEM in hypoxic and normoxic conditions with 25 nmol/L, 50 nmol/L, or without bortezomib. The incubation was continued for 12 h, 24 h, and 48 h. After this time the medium was removed and the cell layers ware washed with 2 mL of PBS. SA-*β*-galactosidase-positive cells were detected using Senescence Detection Kit (bioVision). Briefly, the cells were fixed with 1 mL of Fixative Solution for 15 minutes at room temperature and washed with 2 mL of PBS. After washing Staining Solutions Mix was added [940 *μ*L of Staining Solution, 10 *μ*L of Staining Supplement, and 10 *μ*L of 20 mg/mL 5-bromo-4-chloro-3-indolyl-*β*-D-galactopyranoside (X-gal)] at pH 6.0. This pH ensures that nonsenescence cells remain unstained. The cells were incubated in these conditions for 12 h at 37°C. After incubation the cells were washed with PBS and stained cultures were viewed under an inverted microscope (Olympus CKX 41) at 200 magnification. Representative fields were photographed. The percentage of SA-*β*-galactosidase positive cells was determined by counting the number of blue cells under bright field illumination, and then the total number of cells in the same field under phase contrast.

### 2.7. Sodium Dodecyl Sulphate/Polyacrylamide Gel Electrophoresis (SDS/PAGE)

The cell cultures were washed with cold PBS and solubilised in 200 *μ*L of passive lysis buffer per well. The lysates were centrifuged at 10,000 ×g, at 4°C, for 10 min. The samples of lysates, containing 20 *μ*g of protein, were subjected to SDS-PAGE, as described by Laemmli [27]. The electrophoresis was run for 40–45 minutes. In each experiment 7.5% polyacrylamide gel and constant current (25 mA) were used.

### 2.8. Immunoblotting

The proteins were transferred to nitrocellulose membranes and then pretreated for 2 h with Tris-buffered saline (TBS) containing 0.05% Tween 20 (TBS-T) and 5% nonfat dry milk, at room temperature. Membranes were probed with a mixture containing anti-ORP150 antibody (1 : 100) or anti-HIF-1*α* (1 : 500) in 5% dried milk in TBS-T, at 4°C, for 16 h. Then the alkaline phosphatase conjugated antibody against mouse IgG (whole molecule) was added at concentration 1 : 2500 in TBS-T for 1 h with slow shaking. The nitrocellulose was washed with TBS-T (five times for 5 min) and exposed to Sigma-Fast BCIP/NBT reagent.

### 2.9. Protein Assay

Protein concentration in lysates of cell layers was determined by the method of Smith et al. [28] using BCA Protein Assay Kit (Thermo Scientific, USA). Bovine serum albumin was used as a standard.

### 2.10. Statistical Analysis

The mean values from three independent experiments ± standard deviations (SD) were calculated. Statistical analysis was performed using Student's *t*-test.

## 3. Results

### 3.1. The Effect of Bortezomib on Viability of Fibroblasts

**Figure 1:**
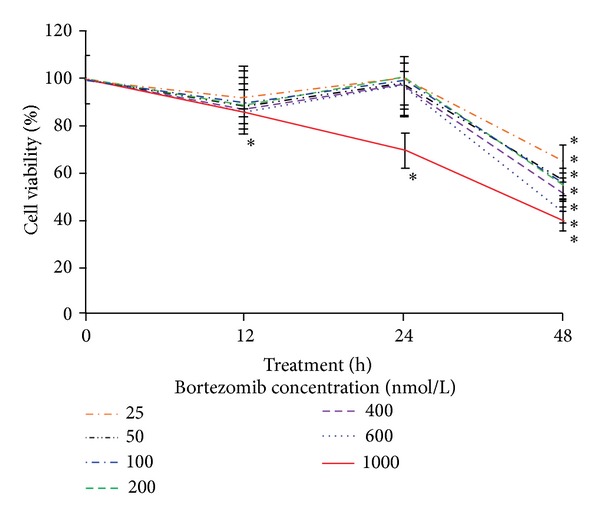
The viability of fibroblasts treated with different concentrations of bortezomib for 12 h, 24 h, and 48 h. The results are mean for pooled triplicate values from three independent experiments. Statistical significance was considered if *P < 0.05.

The antiproliferative effects of bortezomib were assessed by MTT method in fibroblasts cultured with increasing concentrations of bortezomib for periods of 12 h, 24 h, and 48 h.  Figure 1 shows that bortezomib, in the concentration from 25 nmol/L to 1000 nmol/L, caused a time-dependent and dose-dependent reduction in cell viability of the tested cells. An evident inhibition in cell viability was observed as early as 48 h. In cells treated with 1000 nmol/L of bortezomib, the effect on cell viability was markedly more pronounced than in others (Figure 1). Two concentrations of bortezomib (25 nmol/L and 50 nmol/L) were chosen for further study. Both were up to value of the half maximal inhibitory concentration (IC_50_) for bortezomib.

### 3.2. Detection of HIF-1*α* in Fibroblasts Submitted to Hypoxia

**Figure 2:**
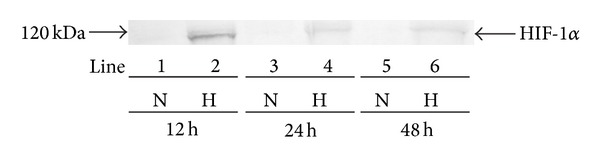
Western immunoblot analysis of HIF-1*α* synthesised by fibroblasts has been presented. The cells were incubated in normoxic and hypoxic conditions for 12 h, 24 h, and 48 h. Samples containing 20 *μ*g of protein were submitted to electrophoresis and immunoblotting.

We also characterised the expression of HIF-1*α*, a biochemical marker of hypoxia.  Figure 2 shows that cells grown in normoxic conditions (for 12 h—lane 1, for 24 h—lane 3 and for 48 h—lane 5) did not demonstrate the expression of HIF-1*α*. In contrast, those cells incubated in hypoxic conditions demonstrated an intense expression of HIF-1*α* after 12 incubation hours (Figure 2, lane 2). The expression of HIF-1*α* after 24 h and 48 h (lane 4 and lane 6) was lower in comparison to 12 h (lane 2).

### 3.3. The Effect of Bortezomib on Apoptosis

**Figure 3:**
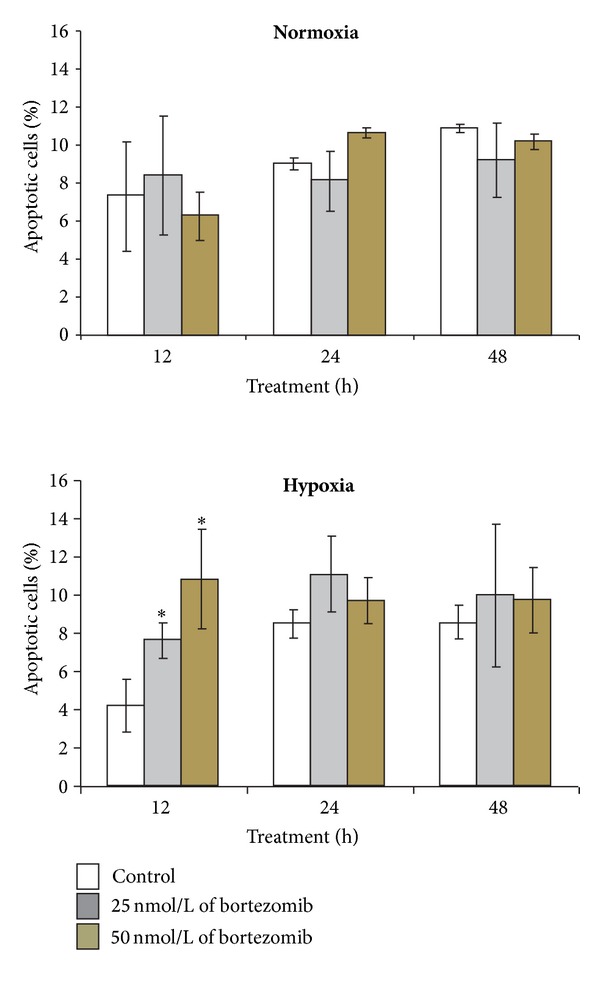
The effect of bortezomib on apoptosis of fibroblasts. The cells were incubated in normoxic and hypoxic conditions for 12 h, 24 h, and 48 h. Mean values from three independent experiments ± SD are presented. Statistical significance was considered if *P < 0.05.

Figure 3 shows the percent of apoptotic fibroblasts in cultures incubated for 12 h, 24 h, and 48 h in normoxic and hypoxic conditions with 25 or 50 nmol/L of bortezomib. We did not observe any effect of bortezomib on apoptosis of fibroblasts in normoxic conditions. The percent of apoptotic cells did not change significantly, independently on incubation time and examined concentration of bortezomib (25 nmol/L or 50 nmol/L). Only in the case of cultures incubated for 12 h in hypoxia with 25 nmol/L of bortezomib we observed 2-fold increase in apoptosis and 3-fold rise with 50 nmol/L of bortezomib in comparison to control cells, incubated without bortezomib. In contrast to 12 h we did not observe any effect of bortezomib on apoptosis of fibroblasts incubated for 24 h and 48 h with or without bortezomib in hypoxic conditions. The percent of apoptotic cells did not change significantly after 24 h or 48 h with 25 nmol/L or 50 nmol/L of bortezomib in comparison to control (Figure 3).

### 3.4. The Effect of Bortezomib on SA-*β*-Galactosidase Expression

The SA-*β*-galactosidase expression was determined with the use of chromogenic X-gal substrate. Normal cells showing traits of division do not undergo reaction with this substrate.

However, the cells with evoked cell division blockade become intensely blue stained. Higher SA-*β*-galactosidase expression in cytoplasm caused the intensification of this reaction.

**Figure 4:**
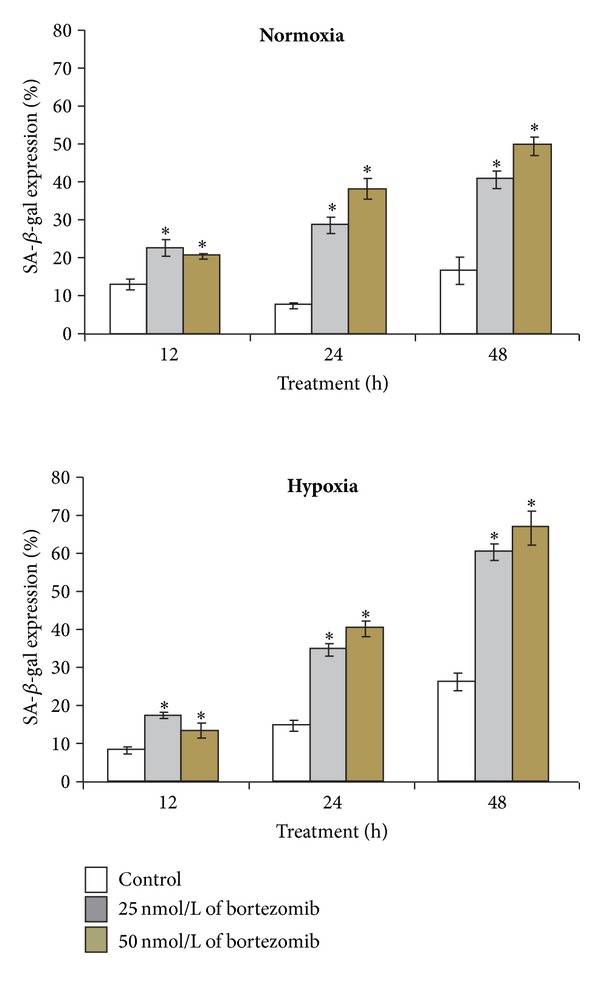
The effect of bortezomib on percentage of SA-*β*-galactosidase expression in fibroblasts. The cells were incubated for 12 h, 24 h and 48 h in normoxic and hypoxic conditions. Mean values from three independent experiments ± SD are presented. Statistical significance was considered if *P < 0.05.

Figure 4 shows the percent of SA-*β*-galactosidase positive fibroblasts incubated for 12 h, 24 h, and 48 h in normoxic and hypoxic conditions with 25 or 50 nmol/L of bortezomib. Bortezomib, in examined concentrations—25 nmol/L and 50 nmol/L, caused a time-dependent increase in senescence of normal fibroblasts (Figure 4). SA-*β*-galactosidase expression was 1.5- to 2.0-fold higher in the cells incubated for 12 h in normoxic conditions with 25 and 50 nmol/L of bortezomib, than in control cells. The prolongation of incubation time up to 24 h resulted in an increase of SA-*β*-galactosidase expression—up to 40% and after 48 h—up to 50%. In hypoxia conditions we observed an increase in senescence of fibroblasts incubated with 25 and 50 nmol/L of bortezomib. The percent of senescence positive cells after 12 h was similar in cultures incubated in hypoxia and normoxia (Figure 4). It is worthy of note that bortezomib exerted very strong effect on senescence of fibroblasts incubated in hypoxic conditions. The SA-*β*-galactosidase expression was raised after 24 h over 40% and after 48 h-over 70%.

**Figure 5:**
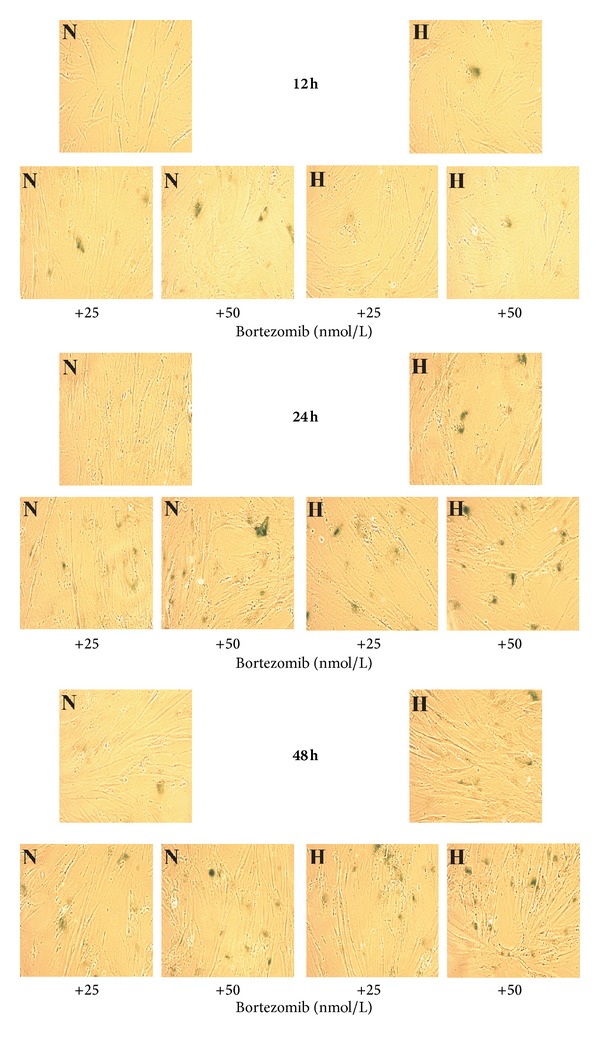
Cytochemical staining for SA-*β*-galactosidase in fibroblasts. The cells were incubated for 12 h, 24 h, and 48 h in normoxic or hypoxic conditions with 25 nmol/L, 50 nmol/L or without bortezomib. A representative photograph (magnification 200x) is presented.

Figure 5 displays representative photographs of positive for SA-*β*-galactosidase staining fibroblasts incubated for 12 h, 24 h, and 48 h in normoxic and hypoxic conditions with 25 nmol/L or 50 nmol/L of bortezomib. Morphological changes of cells play an important role in senescence. After 12 h of cells incubation with 25 nmol/L or 50 nmol/L of bortezomib in hypoxic and normoxic conditions irregular and enlarged cell body shape was observed in comparison with control cells incubated in normoxia and hypoxia without bortezomib. The prolongation of incubation time up to 24 h resulted in a higher number of irregular body shape cells and development of granulation in cytosol, independently of the concentration of bortezomib in comparison to control cells incubated in hypoxia and normoxia without bortezomib. This effect was more evident after 48 h. Additionally, we observed the development of vacuolization in the cytosol of cells incubated in hypoxia conditions only with 50 nmol/L of bortezomib, in comparison to these incubated in hypoxic conditions without bortezomib.

### 3.5. The Effect of Bortezomib on the Expression of ORP150/GRP170

**Figure 6:**
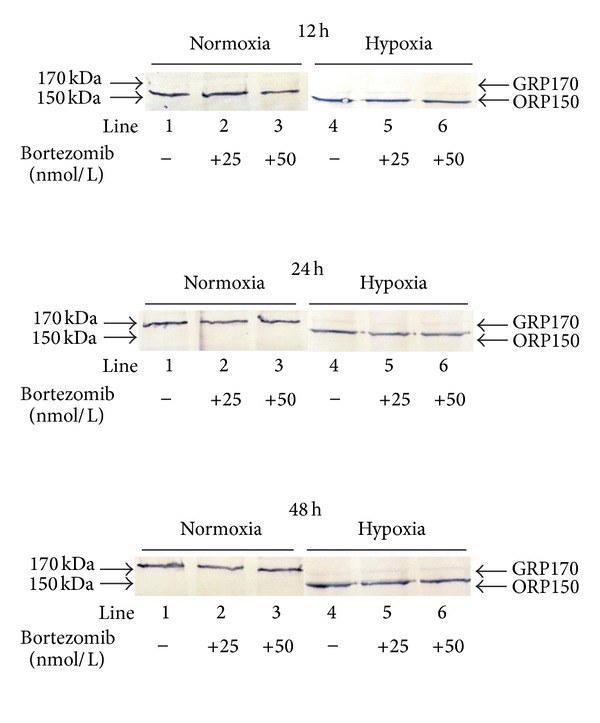
Western immunoblot analysis of ORP150/GRP170 expression in fibroblasts incubated with various concentration of bortezomib in normoxic (lanes: 1–3) and hypoxic (lanes: 4–6) conditions for 12 h, 24 h and 48 h. Lanes 1 and 4—without bortezomib, lanes 2 and 5—with bortezomib—25 nmol/L, lanes 3 and 6—with bortezomib—50 nmol/L. Samples containing 20 *μ*g of protein were submitted to electrophoresis and immunoblotting.

Figure 6 shows the expression of ORP150 and its glycosylated form GRP170 in fibroblasts incubated in normoxic and hypoxic conditions with 25 nmol/L, 50 nmol/L of bortezomib. In hypoxic conditions we observed induction of ORP150 expression. The expression of GRP170 in fibroblasts was observed in cultures incubated in normoxic conditions for 12 h, 24 h, and 48 h with or without bortezomib (Figure 6; lanes 1–3). It is of interest that fibroblasts incubated in normoxia did not express ORP150 (Figure 6; lanes 1–3). In hypoxic conditions we observed the induction of ORP150 expression in fibroblasts incubated for 12 h, with or without bortezomib (Figure 6; lanes: 4–6). Prolongation of incubation up to 24 h and 48 h resulted in intensification of ORP150 expression, especially after 48 h.

## 4. Discussion

The 26S proteasome inhibitor, bortezomib, selectively induces apoptosis in some cancer cells. However, the nature of its selectivity remains unknown. The work presented here provides novel information on cellular effects of bortezomib in normal fibroblasts. We observed that bortezomib slightly reduces cell viability of the tested cultures in a concentration-dependent and time-dependent manner. We have found, that in normoxic conditions the percent of apoptotic cells did not change significantly, independently on incubation time and examined concentration of bortezomib (25 nmol/L or 50 nmol/L). In hypoxic conditions we did not observe any effect of bortezomib on apoptosis of fibroblasts incubated for 24 h and 48 h in comparison to control. Only in the case of fibroblasts incubated for 12 h in hypoxia significant increase in apoptosis, dependent on concentration of bortezomib, was observed. Moreover, we demonstrated that bortezomib, in examined concentrations—25 nmol/L or 50 nmol/L, causes a time-dependent increase in senescence of normal fibroblasts, especially of these incubated in hypoxic conditions. Our findings demonstrated that in contrast to normal fibroblasts, bortezomib treatment evoked strong effect on apoptosis in cancer cells. We observed a time-dependent increase up to 70% in apoptosis of MDA-MB-231 cells, especially these incubated in hypoxic conditions (non published data). Proteasome inhibition have widespread ability to disrupt tumor cell homeostasis and to induce apoptosis *in vitro* in a broad spectrum of tumor cell lines without inducing toxicity in normal cells [29]. There was no effect of bortezomib action on senescence of breast cancer cells (data not shown). In contrast to them, bortezomib in examined concentration caused a time-dependent and dose-dependent increase in senescence of normal fibroblasts. It is worthy of note, that bortezomib exerted very strong effect on senescence of fibroblasts incubated in hypoxic conditions.

The maintenance of protein homeostasis in cell requires the activities of chaperones and the ubiquitin-proteasome system, which together serve to inactivate and degrade misfolded proteins. When proteins are not folded properly, they are directed to 26S proteasomal degradation [10]. If misfolded or unfolded proteins are not degraded by the proteasome, they form aggregates and lead to the ER stress. The ER stress triggers UPR to reduce the accumulation of unfolded proteins and restore the ER function. When protein aggregation or ER stress persists, UPR signaling switches from the prosurvival to proapoptotic [2]. Consequently, the 26S proteasome complex also plays an important role in regulating the ER stress and cell survival [10]. Therefore, inhibition of the proteasomal function in cancer cells would promote apoptosis and have an antitumour function [9]. In fact, the inhibition of the proteolytic activity of the 26S proteasome has been shown to induce proapoptotic ER stress in multiple myeloma [30], pancreatic [31], head and neck cancer [32], and nonsmall cell lung carcinoma [33].

Proteasomal activity is essential for eliminating excess proteins and, by counteracting protein production, establishing steady protein levels [34]. The ubiquitin proteasome pathway represents the major pathway for intracellular protein degradation. The 26S proteasome is responsible for the degradation of approximately 80% of cellular proteins, including misfolded and mutated proteins as well as those involved in the regulation of development, differentiation, cell proliferation, signal transduction, apoptosis, and antigen presentation [8]. Prolonged proteasome inhibition induces stress responses that initiate apoptosis via intrinsic pathway [34]. This is exploited clinically in the treatment of multiple myeloma with the proteasome inhibitor bortezomib. Inhibition of proteasome activity by bortezomib is associated with an accumulation and transcriptional induction of BH3-only proteins such as PUMA, BIM, NOXA, or BIK. BH3-only proteins antagonise antiapoptotic BCL-2 family members such as BCL-2, BCL-xL, or Mcl-1 and can activate the proapoptotic members BAX and BAK [34]. Activated BAX and BAK form pores in the outer mitochondrial membrane, resulting in cytochrome c and Smac/Diablo release from the intermembrane space into the cytosol. This results in caspase-9 activation, inhibition of inhibitor of apoptosis (IAP) proteins, and subsequent apoptosis execution by effector caspases [35]. Induction of NOXA has been reported to be a key mechanism in bortezomib-mediated apoptosis, which is independent on P53 status but dependent on c-Myc [36–39]. Bortezomib-mediated apoptosis is accompanied by induction of c-Jun-NH_2_ terminal kinase, generation of reactive oxygen species, release of cytochrome c, second mitochondria-derived activator of caspases and apoptosis-inducing factor (AIF), and activation of the intrinsic caspase-8 pathway and extrinsic caspase-9 pathway [8].

In agreement with the cytoprotective role of molecular chaperones it has been shown that they can prevent stress-induced apoptotic death [25, 40]. Overexpression of Hsp70 chaperons (ORP150 belongs to this family) prevents cytochrome c release from mitochondria, blocks apoptosome formation by binding to the apoptotic protease-activating factor (Apaf-1), inhibits the release of AIF from mitochondria, and prevents the loss of mitochondrial transmembrane potential. The AIF released from mitochondria binds to Hsp70. This interaction makes impossible the nuclear import of AIF [40].

Little is known about the function of chaperones in nondividing cells. Senescence is thought to play an important role in tumour suppression. Cellular senescence is more than just replicative senescence. It is a common program that is activated by normal cells in response to various types of stress. It has been named “stress-induced premature senescence” [23]. This type of senescence can act as a tumour suppressor in order to prevent damaged cells to multiply as well as a secondary outcome for cancer cells due to therapeutic treatments [18]. Tumour suppressors prevent cells from transforming into cancer by forming molecular barriers for genomic instability and infinite proliferation. They normally induce either apoptosis or a permanent cell cycle arrest-senescence. Unfortunately, it is not known at present which factors determine apoptosis or senescence [18].

Chaperones induction in cancer cells can lead to cancer progression and may be a major cause of chemotherapeutics resistance. It is one of the mechanisms protecting cancer cells against entering the apoptotic pathway. Hence, chaperone inhibition may be a promising tool to decrease cytoprotection and to initiate apoptosis of cancer cells [18]. Senescence and apoptosis normally counteract tumour development and cancer cells must therefore overcome these important tumour suppressor mechanisms to disrupt this barrier. Senescence emerges as an important tumour suppressor mechanism *in vivo*. Understanding and application of cellular senescence for cancer therapy has recently become a field of extensive research [18]. Gabai et al. have shown that knockdown of Hsp72 in certain cancer cells, but not in untransformed breast epithelial cells, triggers senescence via p53-dependent and p53-independent mechanisms. They demonstrate that the p53-dependent pathway controlled by Hsp72 depends on the oncogenic form of phosphatidylinositol 3-kinase (PI3K). Moreover, in cancer cell lines, activation of the p53 pathway caused by depletion of Hsp72 was dependent on oncogenes that activate the PI3K pathway. On the other hand, the p53-independent senescence pathway controlled by Hsp72 was associated with the Ras oncogene. In this pathway, extracellular signal-regulated kinases (ERKs) were critical for senescence, and Hsp72 controlled the ERK-activating kinase cascade at the level of Raf-1. Therefore, Hsp72 is intimately involved in suppression of at least two separate senescence signaling pathways that are regulated by distinct oncogenes in transformed cells, which explains why cancer cells become “addicted” to this heat shock protein [41].

The expression of ORP150 in cultured human cells is essential for their survival under hypoxia [42]. Such data suggest that ORP150 contributes in the cellular response to environmental stress. Our experiments have shown that ORP150 expression was induced in fibroblasts in hypoxia conditions only, suggesting that this protein may play an important role in the cytoprotective response to environmental stress.

At present, the exact signaling mechanism underlying bortezomib-induced apoptosis and senescence is poorly understood. Our study has shown that bortezomib causes a time-dependent increase in senescence of normal fibroblasts, especially of these incubated in hypoxic conditions. Moreover, we demonstrated that oxygen regulated protein 150 (ORP150) expression was induced in fibroblasts in hypoxia conditions only, suggesting that this protein may play an important role in the cytoprotective response to environmental stress. Further investigation of the protective responses will elucidate the mechanism by which fibroblasts are resistant on proteasome inhibition-mediated apoptosis and are directed for senescence.
